# Influence Mechanism on Supplier Emission Reduction Based on a Two-Level Supply Chain

**DOI:** 10.3390/ijerph182312439

**Published:** 2021-11-26

**Authors:** Lina Ma, Xinran Zhang, Yushen Du

**Affiliations:** Business School, Jilin University, Changchun 130012, China; zxr21@mails.jlu.edu.cn (X.Z.); duys@jlu.edu.cn (Y.D.)

**Keywords:** supply chain, emissions reduction, downstream firm’s monitoring, taxation, governmental subsidy

## Abstract

The purpose of this paper is to investigate environmental performance of a supply chain which consists of an upstream supplier and a downstream firm. A mathematical model considering both downstream firm’s monitoring and governmental intervention is developed. Afterwards, a numerical example is presented to show the equilibriums of these models and the optimal choices of firms and government. The results show that when customers’ environmental awareness increases, both total environmental impact and social welfare decrease. The downstream firm’s monitoring will certainly reduce the total environmental impact. In most cases, it does not matter whether the downstream firm chooses to monitor the supplier or not, the total environmental impact and social welfare would not be affected when the government chooses subsidy. If a subsidy is present, firms and environment will be better than those without subsidy. Hence, the government is more likely to choose to provide subsidy and the downstream firm will not monitor the supplier’s greenhouse gas (GHG) emissions reduction effort. In a few cases when environmental impact is too large, taxation may be the optimal choice for the government and the downstream firm will choose to monitor the supplier’s GHG emissions reduction investment.

## 1. Introduction

Increasingly stringent environmental regulations and environmental protection pressure from the public have forced the supply chain terminal enterprises to pay more attention to their suppliers’ performance, and implement green supply chain management. Wal Mart attaches importance to sustainable development and is committed to helping suppliers reduce carbon emissions. Wal Mart has proposed a global “1 billion ton emission reduction project” to join hands with suppliers to reduce greenhouse gas emissions in the global business value chain by 1 billion tons by 2030. Among them, Wal Mart officially launched the global “1 billion tons emission reduction project” in China in 2019 [1]. Apple works closely with its suppliers to fulfill its commitment to the environment step by step. Apple has pledged to be carbon neutral throughout its supply chain by 2030, meaning the entire supply chain will be 100 percent carbon neutral. Next, Apple will focus on Foxconn and other manufacturing suppliers, and increase capital support for suppliers to reduce emissions [2].

Suppliers’ environmental performance has been paid more and more attention by enterprises, and methods for selecting the best supplier in consideration of environmental standards are proposed [3]. Stackelberg game method and other methods are used to study and analyze the benefits of retailers’ low-carbon investment on manufacturers and the whole supply chain [4]. The main finding is that low carbon investment by retailers is beneficial. Through realistic exploration, it is concluded that the company can be more competitive in the market by adopting green supply chain practices [5,6,7,8,9]. The existence of supply chain coordination contract has an incentive effect on the improvement of environmental performance of suppliers and downstream enterprises [10,11,12]. The study of consumers’ preference for low carbon driven by the reduction of carbon emissions is focused on [13]. Consumers’ concern about corporate environmental performance and the demand for sustainable products can drive the sustainable production of enterprises [14,15,16,17,18].

In view of China’s commitment to carbon emissions in 2020, China’s per unit GDP carbon dioxide emissions fell 40–45% from the year of 2005, the government and enterprises are facing different levels of pressure and challenges [19]. In order to better protect the environment, on the one hand, some governments impose taxes on enterprises to punish them for their environmental pollution. On the other hand, some government subsidies are used to guide enterprises to implement emission reduction, for example, Beijing has introduced “Beijing approach to support the use of cleaner production funds” to guide and support enterprises’ effort to implement cleaner production. Haicang District of Xiamen city has implemented energy-saving emissions reduction fund grants to encourage more enterprises to carry out energy conservation and emissions reduction [20,21]. It can be seen that the government’s policy intervention has an important role in emission reduction.

There is already a large body of work on the low-carbon supply chain and government intervention in the enterprise and government decision-making. The trade-old-for-remanufactured (TOR) model for a scenario of carbon tax and government subsidies is established and found that in the absence of carbon tax constraints, the improvement of consumers’ environmental awareness may lead to the increase of enterprises’ carbon emissions [22]. An economic model is developed to study the impact of the environmental awareness of consumers, the taxes and competition on corporate emissions reduction decisions [23,24,25]. The effective implementation of green supply practices in the textile and garment supply chain requires the joint efforts of green technology innovation, consumer awareness, and government regulatory agencies [26,27]. Considering a supply chain of a manufacturer and a retailer, when consumers are sensitive to the environmental profile of the manufacturer’s products, the channel structure of the supply chain members is different, and the cooperation between retailers and manufacturers to promote the green innovation of the manufacturer’s products, the equilibrium decision is compared [28,29]. Manufacturers meet consumers’ green requirements through innovative processes and provide guidance for supply chain managers [30]. Including an upstream supplier and a downstream enterprise, it is considered who the holder of the responsibility should be and have the right to provide the contract in the supply chain [31]. But these studies do not discuss the government’s decision-making.

A few scholars discuss the government subsidy for the green innovation of duopoly, and the Optimal Subsidy Policy of the government and the corresponding management decision of the manufacturer in dealing with government subsidy [32,33,34,35,36]. Some papers discuss the government subsidies to producers for remanufacturing activities, and environmental taxes [37]. Moreover, they also focused on the implementation by the downstream enterprise of green strategy, the evolution process of the downstream enterprise operation model under the market mechanism, and analyzed the role of the government [38]. Some other papers discuss how to promote recycling and remanufacturing activities through subsidies and tax incentives under the extended producer responsibility system [39,40,41]. Some scholars study the effectiveness of Sweden’s carbon tax using an econometric method and notice that the use of a carbon tax alone has a small effect on carbon dioxide emissions in Sweden, except for gasoline [42,43,44].

Subsidies for green innovation funded by carbon tax revenues can significantly reduce carbon emissions [45]. But there are limited discussion about the emissions reduction behavior of the producers and the cooperation or supervision between the members of the supply chain. The effects of government subsidies, carbon taxes, and cost-sharing contracts for energy conservation and emission reduction of upstream and downstream enterprises on supply chain coordination are discussed [46]. In addition, this paper does not discuss the impact of the relationship between government and consumers on environmental performance, as other scholars have done the relevant research. The impact of consumer subsidies on green technology innovation of automobiles and the environmental impact are studied, and the conclusion is that consumer subsidies have a positive impact on the environment [47,48].

In view of the above, to make the research purpose more tangible, this paper uses the model and takes into account the impact of the high cost of pollution in the downstream enterprises, the monitoring of the downstream enterprises and government intervention on the supplier’s decision-making, enterprise profits, ecological environment and social welfare. First, a supply chain model without downstream firm’s monitoring and governmental intervention is set up. Based on the maximum profit of firms, optimal equilibriums are obtained. Second, when considering the downstream firm’s monitoring, a Nash bargaining model on the supplier’s emissions reduction level per unit of output produced is set up for the supplier and downstream firms. Third, when considering governmental intervention, a penalty tax may be imposed on the suppliers to force them to substantially reduce GHG emissions. Or an incentive subsidy may be provided for the supplier to encourage more emissions reduction. To solve the equilibrium decision of the government in maximizing social welfare, the environmental impact of supplier’s production emissions is included in the social welfare function. Finally, the model of considering both downstream firm’s monitoring and governmental intervention is developed. A numerical example is presented to show the equilibriums of these models and the optimal choices of firms and government. This paper will assist government and enterprises in formulating relevant strategies by analyzing the impact of downstream enterprise monitoring and government intervention on supplier emission reduction.

## 2. Model Building and Basic Assumptions

Consider the cost of the supplier’s production unit product *c*; the supplier first gives the wholesale price of the downstream business *p_c_*. Downstream enterprises sell the products to consumers with the retail price of *p*. If consumers are sensitive not only to the retail price; the negative impact on the environment in the production process of the supply chain will also be perceived by consumers and have negative effects on consumer demand [23]. The market demand function of downstream enterprises is:

(1) $$D(p,\Delta e)=a-p-b(e_{0}-\Delta e)$$

Because whether the consumer is sensitive to the retail price of the product is not the focus of this study, therefore, in order to simplify the calculation, assuming that the retail price sensitivity coefficient is 1 [49]. 

*a* is the total potential market demand which cannot be ignored to discuss the impact of consumers’ environmental awareness on product demand, nor to ignore the role of market scale on carbon emissions in the supply chain. 

*b* is consumer awareness of environmental protection, the bigger its value, the more sensitive the consumer is to the supplier’s carbon emissions, when *b* = 0, consumers do not care about the negative impact of carbon emissions by suppliers, therefore, when *b* = 0 suppliers will not reduce emissions through investment.

*e*_0_ represents the supplier’s initial unit carbon emissions, Δ*e* represents the unit production of carbon emissions that the supplier reduces through inputs, which include production technology research and development, investment in cleaner production technology or staff training. So, (*e*_0_ − Δ*e*) represents the quantity of pollutants discharged per unit of output by supplier that the consumer can perceive in the end.

Make *T* the total carbon emissions, then *T* = (*e*_0_ − Δ*e*)*D*(*p*,Δ*e*). Assume that the total cost of the supplier’s commitment to emission reduction is *k*(Δ*e*)^2^,which means that more energy is required for more emissions reductions. This paper does not consider the supplier can complete reduction, because this is inconsistent with the facts; in reality, often companies are forced to shut down because of serious pollution by the government, therefore, it is assumed that the input cost coefficient is large enough, so that the optimal output of the supplier is always less than the initial discharge, and there exists a unique optimal solution for each stage of the equilibrium solution [23]. 

Similar to some existing studies, we assume that the input does not change the marginal cost of the enterprise [23,28]. In addition, we consider investment in emissions reduction or investment in environmental technology innovation literature, set emissions reduction investment as a one-time investment, and only consider the single cycle model, so the cost sharing problems (such as multi cycle, time cost, etc.,) of the entire cycle will not be considered. Since emissions reductions require longer lead times than price setting, it is assumed that the order is: in the first stage the supplier determines the unit displacement Δ*e*, in the second stage supplier determines the wholesale price *p_c_*, in the third stage the downstream enterprises determines the retail price *p*.

## 3. Model Analysis

The supplier emission reduction model will be established and analyzed in the following three cases, that is, without government subsidy taxation and without supervision of downstream enterprises, without government subsidy taxation but with supervision of downstream enterprises, and considering government subsidy or taxation.

### 3.1. Initial Conditions

First, the profit function of downstream enterprises and suppliers is:

(2) $$\pi_{c}=(p_{c}-c)\;(a-p-b\;(e_{0}-\Delta e))-k\;(\Delta e{)}^{2},\;\pi_{m}=(p-p_{c})(a-p-b\;(e_{0}-\Delta e))$$

The subscript *m* represents the downstream enterprise, the subscript *c* represents the supplier. 

Using the backward induction method to solve the model, we can get the optimal pricing of the downstream enterprises and suppliers:

(3) $$p=\frac{1}{2}\;(a-be_{0}+\Delta b+e+p_{c}),\;p_{c}=\frac{1}{2}(a-be_{0}+b\Delta e+c)$$

Once again, the optimal unit discharge:

(4) $$\Delta e=\frac{b\left(a-be_{0}-c\right)}{8k-b^{2}}$$

In order to ensure that the company’s emission reduction is greater than 0, assuming 8*k* − *b*^2^ > 0, *e*_0_ < $\frac{a-c}{b}$, therwise, the enterprise will be eliminated from the market. The carbon emissions per unit of output are changed by the supplier’s unilateral emission reduction efforts:

(5) $$e_{0}-\Delta e=\frac{8ke_{0}-ab+bc}{8k-b^{2}}$$

At this point, the total carbon emissions is:

(6) $$T=(e_{0}-\Delta e)\;(a-p-b(e_{0}-\Delta e))=\frac{2k\left(a-be_{0}-c\right)\left(8ke_{0}-ab+bc\right)}{{\left(8k-b^{2}\right)}^{2}}$$

**Proposition** **1.** *In the initial case, when $e_{0}\;<\;\bar{e_{0}}$, with an increase in e_0_, the total negative impact of carbon emissions on the environment increased; when $\bar{e_{0}}\;<\;e_{0}\;<\;\frac{a-c}{b}$, with an increase in e_0_, the overall negative impact of carbon emissions on the environment is reduced. Furthermore, $\bar{e_{0}}$ = $\frac{\left(8k+b^{2}\right)\left(a-c\right)}{16bk}$.*

**Proof.**  Slightly. □

From (5) we can see that with the increase of *e*_0_, the supplier’s unit carbon emissions increased after emissions reduction, and ultimately the market demand reduced, because there is no other external pressure, the only power of enterprises emissions reduction is to keep the demand, so when *e*_0_ is at a low level, pollution is not too serious. The negative effects that carbon emissions brought on demand are not very obvious. Enterprises lack the power of emissions reduction through investment, making the unit emissions of pollutants increase faster than the rate of decline in market demand. So, with the increase in *e*_0_, the total negative impact on the environment caused by emissions is increasing. When *e*_0_ is increased too much (more than 0), pollution reduced market demand significantly, reducing enterprise’s orders, and the market demand reduced faster than the rate of the increase in enterprises pollution emissions. So in the case of high pollution, with the increase in *e*_0_, the total negative impact on the environment caused by production reduces.

### 3.2. Downstream Enterprises Urged Suppliers to Reduce Emissions

Considering the supplier’s production process cannot be controlled directly by the upstream and downstream enterprises, and the supplier’s pollution will directly affect consumer demand for products of downstream enterprises, it has negative impact on profits and corporate social image. Therefore, in order to meet the demands of consumers, improve the environmental quality of supply chain and corporate social image, and enhance the market demand and downstream business profits, downstream companies often monitor suppliers to increase the intensity of pollution reduction in the production process. Consider the relationship between the downstream enterprises and suppliers to discuss the reduction of unit carbon emissions [28], which follows the Nash bargaining game as:

(7) $$\underset{e}{Max}\;\pi_{\mathrm{mc}}=\underset{e}{Max}\;\pi_{m}\pi_{c}$$

Function indicates that when the downstream enterprise monitors the supplier, the downstream enterprise only pays attention to the change of the supplier’s unit emissions, rather than sharing the cost of this.

The optimal solution of Δ*e* obtained by backward induction is:

(8) $$\Delta e=\frac{\left(2\sqrt{k^{2}+kb^{2}}-2k+b^{2}\right)\left(a-be_{0}-c\right)}{b\left(8k-b^{2}\right)}$$

When compared to no downstream companies to urge, after the discussion of the supplier’s unit displacement has indeed been improved, the market demand has been improved, the total carbon emissions is:

(9) $$T^{b}=(e_{0}-\Delta e)\;(a-p-b(e_{0}-\Delta e))\;\frac{\left(\left(6k+2\sqrt{k^{2}+kb^{2}}\right)be_{0}-\left(a-c\right)\left(2\sqrt{k^{2}+kb^{2}}-2k+b^{2}\right)\right)\left(3k+\sqrt{k^{2}+kb^{2}}\right)\left(a-be_{0}-c\right)}{2b{\left(8k-b^{2}\right)}^{2}}$$

Comparison of the differences in the total emissions between whether the downstream enterprises urge to get:

(10) $$T^{b}-T=\frac{1}{2b{\left(8k-b^{2}\right)}^{2}}\left(\left(20k^{2}+2kb^{2}+12k\sqrt{k^{2}+kb^{2}}-32k^{2}b\right)e_{0}-\left(kb^{2}+\left(4k+b^{2}\right)\sqrt{k^{2}+kb^{2}}-4k^{2}\right)\left(a-c\right)\right)\left(a-be_{0}-c\right)$$

**Proposition** **2.** *Under the given conditions of other exogenous variables, when $e_{0}\;>\;\tilde{e_{0}}$, T^b^ − T > 0; conversely, T^b^ − T < 0. Moreover,*

$$\tilde{e_{0}}=\frac{\left(kb^{2}+\left(4k+b^{2}\right)\sqrt{k^{2}+kb^{2}}-4k^{2}\right)\left(a-c\right)}{20k^{2}+2kb^{2}+12k\sqrt{k^{2}+kb^{2}}-32k^{2}b}.$$

**Proof.**  Slightly. □

Proposition 2 shows that although the downstream firm’s drive to reduce the supplier’s carbon emissions per unit, it does not fully ensure that the supplier’s total emissions are reduced. As compared to the initial situation, bargaining behavior increased the level of Δ*e*. The unit carbon emissions after emission reductions are reduced under the condition of *e*_0_, but because the emission reduction measures stimulate the demand to a certain extent, when the supplier is a heavy polluter(*e*_0_ > 0), the urging behavior of downstream firms stimulates market demand and increases the production order of the firm, indirectly increasing the total carbon emissions. At the same time due to the difficult governance of heavily polluting enterprises (even if Δ*e* increases, the high level of *e*_0_ will still raise the level of unit emissions after emission reduction), even if the downstream enterprises monitor and urge pollution control, it may not necessarily reduce the negative impact of production emissions on the environment.

### 3.3. Consider Government Intervention

When the government considers to adopt subsidy or tax policies, this paper is divided into four scenarios to analyze, namely, the government taxation model without supervision of downstream enterprises, the government subsidy model without supervision of downstream enterprises, the government taxation model with supervision of downstream enterprises, and the government subsidy model with supervision of downstream enterprises.

#### 3.3.1. Government Taxation

In view of the increasing impact of economic development on the natural environment, low-carbon economy has received growing attention, “Twelfth Five-Year Plan for the Control of Greenhouse GHG Emissions” adopted by the State Council in November 2011 is a sign that China’s low-carbon development has entered the stage of action. The government has the right to impose an environmental tax on the output of the polluting enterprises in order to punish the negative impact on the environment [37]. Therefore, in this context, assuming that the government imposes a tax *m* on the unit output of the supplier [37]. When the government is involved, assuming that the first stage is decided by the government, in the second stage the supplier decides the unit emissions reduction D*e*, in the third stage the supplier decides the wholesale price *p_c_*, in the fourth stage downstream enterprise determines the retail price *p* [32]. When the government levies, the downstream business’s profit function is unchanged, the profit function of the supplier is:

(11) $$\pi_{c}=(p_{c}-c-m)\;(a-p-b(e_{0}-\Delta e))-k(\Delta e{)}^{2}$$

Through the backward induction method, we can obtain the optimal unit discharge:

(12) $$\Delta e=\frac{b\left(a-be_{0}-c-m\right)}{8k-b^{2}}$$

As the government’s job is maximization of social welfare, the government cannot be biased toward the interests of a particular sector of the community, but should consider the impact of decision-making on all sectors of society and groups [37]. The government regardless of the adoption of tax or subsidy policy always put the overall impact of the production operation on the environment into the scope of social welfare assessment [37]. So, the expression of social welfare is:

(13) $$\underset{m}{Max}\;SW=\pi_{c}+\pi_{m}+\mathrm{CW}+\mathrm{GR}-T\;=\;\pi_{c}+\pi_{m}+\frac{{\left(a-p-b\left(e_{0}-\Delta e\right)\right)}^{2}}{2}\;+m\left(a-p-b\left(e_{0}-\Delta e\right)\right)-s\left(e_{0}-\Delta e\right)\left(a-p-b\left(e_{0}-\Delta e\right)\right)$$

where *CW* is the consumer welfare, *GR* for government tax revenue, *s* for suppliers to produce a total carbon emissions of the negative impact on the environment.

**Proposition** **3.** *When $s<\frac{2k-b^{2}}{2b}$ and $e_{0}>\overset{\vee }{e_{0}}$, the government’s optimal tax revenue is $m=\frac{\left(8sk+6bk+sb^{2}\right)e_{0}-\left(6k+2bs\right)\left(a-c\right)}{2k-b^{2}-2bs}$; when $s<\frac{2k-b^{2}}{2b}$ and $e_{0}\leq \overset{\vee }{e_{0}}$, regardless of whether the supplier to reduce emissions through investment, the government should not be taxed. Moreover, $\overset{\vee }{e_{0}}=\max\{\frac{\left(6k+2bs\right)\left(a-c\right)}{8sk+6bk+sb^{2}},\frac{b\left(a-c\right)}{2k-bs}\}$. If $s>\frac{2k-b^{2}}{2b}$, m does not show equilibrium solution.*

**Proof.**  Calculating the social welfare function can obtain the first and two order derivatives of *m*:

(14) $$\frac{\partial SW}{\partial m}=\frac{2k\left(\left(8sk+6bk+sb^{2}\right)e_{0}-\left(6k+2bs\right)\left(a-c\right)-\left(2k-b^{2}-2bs\right)m\right)}{{\left(8k-b^{2}\right)}^{2}}$$

(15) $$\frac{\partial^{2}SW}{\partial m^{2}}=-\frac{2k\left(2k-b^{2}-2bs\right)}{{\left(8k-b^{2}\right)}^{2}}$$

 □

It can be seen that, when $s>\frac{2k-b^{2}}{2b}$, $\frac{\partial^{2}SW}{\partial m^{2}}>0$, at this time *m* does not reach equilibrium solution, this shows that when the negative impact on the environment of production pollution is particularly serious, the social welfare will increase with the increase of government revenue, which indicates that in serious pollution situation, the government would be willing to risk corporate profits and consumer welfare in order to punish polluters, this is mainly because the environmental improvement benefits increased more than other sectors of society welfare loss.

However, when $s<\frac{2k-b^{2}}{2b}$, $\frac{\partial^{2}SW}{\partial m^{2}}<0$, the only condition for the existence of the only optimal solution is that *m* > 0 and *m* makes *e*_0_ − Δ*e* > 0. When *e*_0_ is large enough, the first derivative of *m* has the unique optimal solution to maximize social welfare. Otherwise, when *e*_0_ is small enough, the first derivative of *m* is always less than 0. With the increase of *m*, social welfare decreases, regardless of whether the supplier reduction behavior, downstream enterprises, and suppliers will add the government’s penalties to the wholesale price and retail price, and ultimately increase consumer costs, reduce consumer welfare, and punitive tax greatly reducing the profits of the enterprise, which is not conducive to industrial development. Because the supplier’s pollution is not serious, the welfare improvement of the environmental protection through taxation is not enough to offset its losses to the industry chain and consumers, therefore, when the supplier is a heavily polluting enterprise, even if it has the emissions reduction behavior, the government may resort to taxation; and when the supplier is lighter, the government should not levy taxes.

#### 3.3.2. Government Subsidies

Although the government has imposed environmental taxes on production, it has been recognized by a number of national and government departments. There are also government subsidies for enterprises to invest in emissions reduction [20,21]. Emissions reduction often requires companies to innovate in production technology, especially cleaner production technology, which often requires a lot of money [23]. Therefore, assumptions are made about government subsidies for technological innovation investment [50], and it is assumed that the government provides a certain percentage of subsidy support for supplier emissions reductions, the subsidy ratio is *f*. At this point the supplier’s profit function is:

(16) $$\pi_{c}=\left(p_{c}-c\right)\left(a-p-b\left(e_{0}-\Delta e\right)\right)-k{\left(\Delta e\right)}^{2}\left(1-f\right)$$

Through the backward induction method, we can obtain the optimal unit discharge:

(17) $$\Delta e=\frac{b\left(a-be_{0}-c\right)}{8k\left(1-f\right)-b^{2}}$$

From the above we can see that the government’s subsidy ratio must be less than 1, otherwise the supplier’s unit emissions reduction is negative. 

At this point the expression of social welfare is:

(18) $$\underset{f}{Max}\;SW=\pi_{c}+\pi_{m}\;+\mathrm{CW}-\mathrm{GE}-T\;\;=\;\pi_{c}+\pi_{m}+\frac{{\left(a-p-b\left(e_{0}-\Delta e\right)\right)}^{2}}{2}-fk{\left(\Delta e\right)}^{2}-s\left(e_{0}-\Delta e\right)\left(a-p-b\left(e_{0}-\Delta e\right)\right)$$

**Proposition** **4.** *If $s<\frac{32k-7b^{2}}{8b}$, when $k<\frac{32bs^{2}+52b^{2}s+21b^{3}}{96b+128s}$ and $e_{2}<e_{0}<e_{3}$, or $k>\frac{32bs^{2}+52b^{2}s+21b^{3}}{96b+128s}$ but $e_{2}<e_{0}<e_{3}$ or $e_{4}<e_{0}<e_{1}$, the optimal subsidy ratio given by the government to the supplier is:*

$$f=\frac{\left(6bk+8sk+sb^{2}\right)\left(a-c\right)-\left(16bsk+6b^{2}k\right)e_{0}}{2k\left(\left(7b+4s\right)\left(a-c\right)-\left(7b^{2}+8bs\right)e_{0}\right)}$$

*In other cases, the f equilibrium solution does not exist. Furthermore,*

$$e_{1}=\frac{\left(6bk+8sk+sb^{2}\right)\left(a-c\right)}{16bsk+6b^{2}k},\;e_{2}=\frac{\left(7b+4s\right)\left(a-c\right)}{7b^{2}+8bs},\;\;e_{3}=\frac{\left(8bk-sb^{2}\right)\left(a-c\right)}{8b^{2}k},e_{4}=\frac{\left(7b+4s\right)\left(a-c\right)}{32k}.$$

**Proof.**  The first and two order derivatives of *f* can be obtained by calculating the social welfare function:

(19) $$\begin{array}{c} \frac{\partial SW}{\partial f}=\frac{1}{{\left(8k\left(1-f\right)-b^{2}\right)}^{3}}(2bk(a-c-be_{0})(\left(6bk+8sk+sb^{2}\right)\left(a-c\right)\\ -\left(16bsk+6b^{2}k\right)e_{0}-2k\left(\left(7b+4s\right)\left(a-c\right)-\left(7b^{2}+8bs\right)e_{0}\right)f)) \end{array}$$

(20) $$\begin{array}{c} \frac{\partial^{2}SW}{\partial f^{2}}=\frac{1}{{\left(8k\left(1-f\right)-b^{2}\right)}^{4}}(4bk^{2}(a-c-be_{0})((16bk+64sk+16sb^{2}+7b^{3}\\ -64skf)\left(a-c\right)+(128skbf+112b^{2}kf-16b^{2}k-8sb^{3}-128skb-7b^{4})e_{0}) \end{array}$$

 □

Since the positive and negative derivatives of the second derivative of *f* cannot be directly observed from Equation (20), so first make $\frac{\partial SW}{\partial f}=0$, the only point to make $\frac{\partial SW}{\partial f}=0$ is $f=\frac{\left(6bk+8sk+sb^{2}\right)\left(a-c\right)-\left(16bsk+6b^{2}k\right)e_{0}}{2k\left(\left(7b+4s\right)\left(a-c\right)-\left(7b^{2}+8bs\right)e_{0}\right)}$, replacing the value back to the second order condition, obtain:

(21) $$\frac{\partial^{2}SW}{\partial f^{2}}=-\frac{4k^{2}{\left(\left(7b+4s\right)\left(a-c\right)-\left(7b^{2}+8bs\right)e_{0}\right)}^{4}}{\left(32k-7b^{2}-8bs\right){\left(\left(32k-7b^{2}-8bs\right)\left(a-c\right)-\left(32bk-8b^{2}s\right)e_{0}\right)}^{2}}$$

It is known that when $s>\frac{32k-7b^{2}}{8b}$, $\frac{\partial^{2}SW}{\partial f^{2}}>0$, there is no equilibrium solution for *f* at this time. Similar to the case of government taxes, when the production of pollution to the environment caused by the negative impact of the environment is particularly serious, improving the environment can increase social welfare. When the case of $s<\frac{32k-7b^{2}}{8b}$ is considered, the equilibrium solution must make the government’s equilibrium subsidy rate 0 < *f* < 1, and the government’s optimal subsidy rate must make the supplier’s emission reduction less than its initial emissions, $e_{0}-\Delta e>0$, that is the case of that there is no government unlimited subsidies to make its emission reductions exceed their initial emissions. Considering the existence of the equilibrium solution of the government subsidy, replace the equilibrium expression of *f* back to Equation (17), it can be obtained by $e_{0}-\Delta e=-\frac{\left(7b+4s\right)\left(a-c\right)-32ke_{0}}{32k-7b^{2}-8bs}$, when $e_{0}>e_{4}$, $e_{0}-\Delta e>0$, while *e*_0_ making 0 < *f* < 1 must meet that $e_{0}<\min\{e_{1},\;e_{2}\}$ and $e_{0}<e_{3}$ or $e_{0}>\min\{e_{1},\;e_{2}\}$ and. A simple comparison can get $e_{4}<e_{2}$ and $e_{1}<e_{2}<e_{3}$, so when $e_{0}<e_{3}$

1. $e_{1}<e_{4}$, that is when $k<\frac{32bs^{2}+52b^{2}s+21b^{3}}{96b+128s}$, the equilibrium solution exists if and only if $e_{2}<e_{0}<e_{3}$.
2. $e_{1}>e_{4}$, that is when $k>\frac{32bs^{2}+52b^{2}s+21b^{3}}{96b+128s}$, the equilibrium solution exists if and only if $e_{4}<e_{0}<e_{1}$.

#### 3.3.3. Tax and Downstream Enterprise Monitoring Urges Parallel

In this case, assuming that the government will impose a tax *g* on the supplier’s output per unit, the supplier’s profit function is:

(22) $$\pi_{c}=\left(p_{c}-c-g\right)\left(a-p-b\left(e_{0}-\Delta e\right)\right)-k{\left(\Delta e\right)}^{2}$$

The process of obtaining the optimal solution for *e* is shown in the Formula (7). Through the backward induction method, we obtain the optimal unit discharge is

(23) $$\Delta e=\frac{\left(2\sqrt{k^{2}+kb^{2}}-2k+b^{2}\right)\left(a-be_{0}-c-g\right)}{b\left(8k-b^{2}\right)}$$

The expression of social welfare is:

(24) $$\underset{g}{Max}\;SW=\pi_{c}+\pi_{m}+\mathrm{CW}-\mathrm{GR}-T\;\begin{array}{c} =\pi_{c}+\pi_{m}+\frac{{\left(a-p-b\left(e_{0}-\Delta e\right)\right)}^{2}}{2}\\ +g\left(a-p-b\left(e_{0}-\Delta e\right)\right)-s\left(e_{0}-\Delta e\right)\left(a-p-b\left(e_{0}-\Delta e\right)\right) \end{array}$$

The first and second order derivatives of *g* can be obtained by calculating the social welfare function:

(25) $$\begin{array}{l} \frac{\partial SW}{\partial g}=-\frac{1}{4b^{2}{\left(8k-b^{2}\right)}^{2}}((22b^{2}k^{2}+20sb^{3}k+5b^{3}k-64k^{3}-16bsk^{2}\\ +(64k^{2}+16bsk+4sb^{3}+2b^{4}-6b^{2}k)\sqrt{k(b^{2}+k)})(a-c)+(k(64k^{2}+26b^{2}k+16bsk-11b^{4}-20sb^{3})\\ +\sqrt{k(b^{2}+k)}(22b^{2}k-64k^{2}-4b^{4}-4sb^{3}-16bsk))g-((2b^{5}+64bk^{2}+32sb^{2}k+2sb^{4}-6b^{3}k)\sqrt{k(b^{2}+k)}\\ +22b^{3}k^{2}+32sb^{2}k^{2}+14sb^{4}k+5b^{5}k-64bk^{3})e_{0}) \end{array}$$

(26) $$\frac{\partial^{2}SW}{\partial g^{2}}=-\frac{1}{4b^{2}{\left(8k-b^{2}\right)}^{2}}(k(64k^{2}+26b^{2}k+16bsk-11b^{4}-20sb^{3})+\sqrt{k(b^{2}+k)}(22b^{2}k-64k^{2}-4sb^{3}-16bsk))$$

**Proposition** **5.** *When $e_{0}<\max\{e_{5},e_{6}\}$, in the case of downstream enterprises to urge suppliers to reduce emissions, the government should not impose a tax on suppliers; when $e_{0}>\max\{e_{5},\;e_{6}\}$, the government’s optimal tax amount is $g=\overset{\wedge }{g}$, and*

(27) $$e_{5}=\frac{\left(22b^{2}k^{2}+20sb^{3}k+5b^{4}k-64k^{3}-16bsk^{2}+(64k^{2}+16bsk+4sb^{3}+2b^{4}-6b^{2}k)\sqrt{k(b^{2}+k)})(a-c\right)}{22b^{3}k^{2}+32sb^{2}k^{2}+14sb^{4}k+5b^{5}k-64bk^{3}+(2b^{5}+64bk^{2}+32sb^{2}k+2sb^{4}-6b^{3}k)\sqrt{k(b^{2}+k)}}$$

(28) $$e_{6}=\frac{\left(10b^{3}k-8bk^{2}+(8bk+2b^{3})\sqrt{k(k+b^{2})})(a-c\right)}{64k_{3}+18b^{2}k^{2}-b^{4}k+8bsk^{2}-10sb^{3}k-(2b^{4}+8bsk+64k^{2}+2sb^{3}-30b^{2}k)\sqrt{k(k+b^{2})}}$$

(29) $$\hat{g}=\frac{\begin{array}{l} (22b^{3}k^{2}+32sb^{2}k^{2}+14sb^{4}k+5b^{5}k-64bk^{3}+(2b^{5}+64bk^{2}+32sb^{2}k+2sb^{4}-6b^{3}k)\sqrt{k(b^{2}+k)}e_{0}-\\ \left(22b^{2}k^{2}+20sb^{3}k+5b^{4}k-64k^{3}-16bsk^{2}+(64k^{2}+16bsk+4sb^{3}+2b^{4}-6b^{2}k)\sqrt{k(b^{2}+k)})(a-c\right) \end{array}}{k(64k^{2}+26b^{2}k+16bsk-11b^{4}-20sb^{3})+\sqrt{k(b^{2}+k)}(22b^{2}k-64k^{2}-4b^{4}-4sb^{3}-16bsk)}$$

#### 3.3.4. Government Subsidies and Downstream Enterprise Monitoring

In this case, it is assumed that the government’s subsidy rate to the supplier is *h*, and the supplier’s profit function is

(30) $$\pi_{c}=\left(p_{c}-c\right)\left(a-p-b\left(e_{0}-\Delta e\right)\right)-k{\left(\Delta e\right)}^{2}(1-h)$$

The process of obtaining the optimal solution for Δ*e* is still shown in the Formula (7). Through the backward induction method, we obtain the optimal unit discharge:

(31) $$\Delta e=\frac{\left(2\sqrt{kb^{2}\left(1-h\right)+k^{2}{\left(1-h\right)}^{2}}-2k\left(1-h\right)+b^{2}\right)\left(a-be_{0}-c\right)}{b\left(8k\left(1-h\right)-b^{2}\right)}$$

At this point the expression of social welfare is:

(32) $$\begin{array}{cc} \underset{h}{Max}\;SW=\pi_{c}+\pi_{m}+\mathrm{CW}-\mathrm{GE}-T & =\pi_{c}+\pi_{m}+\frac{{\left(a-p-b\left(e_{0}-\Delta e\right)\right)}^{2}}{2}\\ & -kh{\left(\Delta e\right)}^{2}-s\left(e_{0}-\Delta e\right)\left(a-p-b\left(e_{0}-\Delta e\right)\right) \end{array}$$

By calculating the first derivative of the social welfare function on *h*, the only point that makes $\frac{\partial SW}{\partial h}=0$ is $h^{*}$.

**Proposition** **6.** *When $h^{*}$ makes $\frac{\partial^{2}SW}{\partial h^{2}}<0$, 0 < f < 1 and $e_{0}-\Delta e>0$, $h^{*}$ is the government’s optimal subsidy ratio, otherwise the government will not subsidize the reduction of investment suppliers.*

**Proof.**  Slightly. □

## 4. Numerical Analysis

Considering the complexity of the listed, using Maple software as the calculating tool to obtain approximate solutions for each formula, through the numerical analysis, the paper analyzes the enterprise profit, the negative impact of the production on the environment, and the total social welfare under different circumstances in order to get useful conclusions to provide a reference for the relevant government departments and supply chain enterprise decision-making. Because of the different parameters, the government’s decision-making is different, so different parameters are used for comparative analysis. This paper uses the basic parameter *a* = 5000, *c* = 200, *k* = 4, *s* = 1, makes *b* {0.2,0.4,0.6,0.8}, *e*_0_ {2200,2400,2600,2800} to represent the different levels of consumer awareness of environmental protection and initial unit emissions by suppliers.

Since government’s decision-making precedes the enterprise’s, the government’s decision-making must anticipate the decision-making of enterprises. It is seen from Table 1 and Table 2, the downstream enterprises urging suppliers to reduce emissions can always increase the profits of the downstream business, but will reduce the profits of suppliers. If the downstream enterprises cannot provide enough subsidies to suppliers, suppliers and downstream enterprises will never reach an agreement. Especially when the government chooses the subsidy model, whether the downstream enterprise urges the supplier to reduce emissions does not affect the profit of the downstream enterprise. If the downstream enterprise urges the supplier to reduce emissions, the profit of the supplier will be greatly reduced, and the downstream enterprise cannot provide the funds to subsidize the supplier, so when the government chooses to subsidize the supplier, the downstream enterprise will not urge the supplier to reduce emissions.

When the government chooses the tax model, it can be seen from the numbers 11 and 14 that the downstream enterprises will not urge the suppliers to reduce emissions, and from the combination of the numbers 12 and 15, downstream companies’ urging suppliers to reduce emissions will increase the profits of upstream and downstream enterprises at the same time. Downstream enterprises and suppliers can reach an agreement, at this time the downstream enterprises will always urge the supplier to reduce emissions. The downstream business selection behavior is shown in Table 3.

As can be seen from Table 4, the stronger the consumer awareness of environmental protection, the lower the negative impact on the environment, but also the lower the social welfare. Downstream corporate oversight does reduce the negative impact of supplier production on the environment. In most cases, regardless of whether the downstream companies have urged suppliers to reduce emissions, government subsidies do not have an influence on the negative impact of supplier production on the environment and social welfare, and always better than when there is no government intervention, the government will choose the subsidy model. However, under the combination of parameters 11, 12, 14, and 15, it can be seen that government taxation is more favorable to the environment and is always better than other cases, only from the perspective of protecting the environment. 

As shown in Table 5, from the perspective of social welfare, only in the combination of numbers 12 and 15, the government tax is better than government subsidies. Moreover, when government taxation and downstream corporate supervision urge are implemented together, there would be greater social welfare. As mentioned earlier, under the combination of numbers 12 and 15, the downstream companies will always urge the supplier to reduce emissions, and suppliers and downstream companies can reach an agreement, and at this point the government will choose the tax model.

However, under the combination of numbers 11 and 14, downstream firms will not urge suppliers to reduce emissions, and the government’s choice will depend on the focus of government work. When the government is determined to tackle environmental pollution, it will prefer the taxation model. When the government wants to solve the problem of environmental pollution while taking into account the industrial development and consumer welfare, the government will tend to choose the subsidy model. In the combination of other numbers, regardless of whether the downstream companies have urged suppliers to reduce emissions, the negative impact of production on the environment and social welfare is always consistent under the government subsidy model. It is always better than other circumstances, so the government will choose the subsidy model. Therefore, considering the environmental impact and social welfare and other factors, the government’s choice is shown in Table 6.

In the case of the best choice between government and downstream companies in Table 3 and Table 6, it can be seen that in most cases the government will choose to subsidize suppliers and downstream companies will not urge suppliers to reduce emissions; under the combination of numbers 12 and 15, the government will choose to tax the supplier, and downstream companies will urge suppliers to reduce emissions; in the number 11 and 14 combination, regardless of if the government chooses the tax or subsidy model, the downstream companies will not urge suppliers to reduce emissions.

## 5. Discussion

The existing literature and findings have focused on the low-carbon supply chain and government intervention in the enterprise and government decision-making, but there are limited discussion about the emissions reduction behavior of the producers and the cooperation or supervision between the members of the supply chain. To analyze the impact of downstream enterprise monitoring and government intervention on supplier emission reduction, Nash equilibrium model and mathematical models proposed in this study provide a comprehensive consideration about the downstream enterprises urging suppliers to increase emissions reductions, the government of the supplier of production pollution tax incentives or subsidies for suppliers to subsidize when consumers disapprove of supply chain emissions.

It should be noted that this paper mostly belongs to the decision-making at the strategic level of enterprises, and the model does not consider various uncertainties and information asymmetry in the actual operation process, such as the uncertainty of demand, the uncertainty of output, and the recycling time of waste products. Considering these uncertainties in future research, we will make the supply chain model more realistic and better guide practice. In addition, this paper considers the relatively simple supply chain structure and the small number of upstream and downstream. In fact, the supply chain network is very complicated. A manufacturer may have multiple suppliers and distributors and face fierce peer competition. So, one of the research directions in the future will be considering horizontal competition effect on the low carbon supply chain management.

## 6. Conclusions

We conclude under what conditions the government should choose tax or subsidy, and the negative impact of production emissions on the environment into the social welfare calculation, the model of optimal environment and total social welfare when the consumer awareness of environmental protection and the degree of pollution of the supplier is different compared by numerical analysis.

The analysis shows that under the lack of government intervention, the downstream enterprises’ urging suppliers to reduce emissions is always able to enhance the profits of downstream enterprises, but will reduce the profits of suppliers. Only when the downstream enterprises give suppliers a certain amount of subsidies, business and downstream companies will agree on the extent of the reduction. When the government is involved, in most cases, the government will choose to subsidize suppliers, and downstream companies will not urge suppliers to reduce emissions. In a few cases, the government will choose to tax suppliers, while downstream companies will also urge suppliers to reduce emissions. This paper also provides a certain basis for the formulation of government policies. The government can formulate different policies according to different enterprises, distinguish between different situations, and choose taxes or subsidies to reduce pollution.

From the perspective of environmental protection, government taxation is more beneficial to the environment than government subsidies, and it is always better than other situations. In terms of consumer welfare and business economic development, government subsidies are more beneficial than taxation. Therefore, the government’s choice depends on its priorities. Once the government made up its mind to give priority to environmental pollution, they tend to choose taxation. However, when the government wants to solve the problem of environmental pollution while taking into account industrial development and consumer welfare, they tend to choose subsidies.

## Figures and Tables

**Table 1:** Profit of downstream enterprises.

Number	b	e_0_	Initial Situation	Downstream Enterprises Urged	Government Tax	Government Subsidy	Downstream Enterprise Urged + Tax	Downstream Enterprise Urged + Subsidies
1	0.2	2200	1,191,076	1,194,048	\	1,207,002	\	1,207,002
2	0.2	2400	1,169,321	1,172,239	\	1,184,804	\	1,184,804
3	0.2	2600	1,147,768	1,150,632	\	1,162,813	\	1,162,813
4	0.2	2800	1,126,414	1,129,225	\	1,141,028	\	1,141,028
5	0.4	2200	970,077	979,706	\	997,414	\	997,414
6	0.4	2400	930,886	940,128	\	956,499	\	956,499
7	0.4	2600	892,503	901,362	\	916,441	\	916,441
8	0.4	2800	854,928	863,414	\	877,239	\	877,239
9	0.6	2200	774,222	791,361	\	808,032	\	808,032
10	0.6	2400	721,748	737,725	\	751,839	\	751,839
11	0.6	2600	671,115	685,971	632,074	697,671	\	697,671
12	0.6	2800	622,324	636,100	158,019	645,527	207,395	645,527
13	0.8	2200	601,416	624,801	\	636,568	\	636,568
14	0.8	2400	539,775	560,766	444,445	568,664	\	568,664
15	0.8	2600	481,467	500,189	27,778	504,643	39,550	504,643
16	0.8	2800	426,489	443,074	\	444,444	\	444,444

**Table 2:** Profit of suppliers.

Number	b	e_0_	Initial Situation	Downstream Enterprises Urged	Government Tax	Government Subsidy	Downstream Enterprise Urged + Tax	Downstream Enterprise Urged + Subsidies
1	0.2	2200	2,349,174	2,376,215	\	2,395,027	\	2,376,347
2	0.2	2400	2,335,720	2,332,815	\	2,351,132	\	2,332,942
3	0.2	2600	2,292,666	2,289,814	\	2,307,644	\	2,289,937
4	0.2	2800	2,250,013	2,247,214	\	2,264,561	\	2,247,332
5	0.4	2200	1,930,452	1,920,988	\	1,957,464	\	1,921,474
6	0.4	2400	1,852,462	1,843,381	\	1,877,775	\	1,843,825
7	0.4	2600	1,776,080	1,767,373	\	1,799,741	\	1,767,778
8	0.4	2800	1,701,307	1,692,966	\	1,723,364	\	1,693,333
9	0.6	2200	1,531,024	1,514,534	\	1,564,097	\	1,515,367
10	0.6	2400	1,427,257	1,411,884	\	1,456,705	\	1,412,578
11	0.6	2600	1,327,130	1,312,836	1,249,927	1,353,132	\	1,313,402
12	0.6	2800	1,230,645	1,217,390	312,482	1,253,378	396,921	1,217,837
13	0.8	2200	1,178,770	1,156,916	\	1,212,678	\	1,157,801
14	0.8	2400	1,057,959	1,088,340	871,111	1,085,902	\	1,038,924
15	0.8	2600	943,673	926,174	54,445	966,120	73,232	926,495
16	0.8	2800	835,918	820,417	\	853,333	\	820,513

**Table 3:** The choice of downstream enterprises under different government policies.

Government Policy	Downstream Enterprises
Subsidies to suppliers	Will not urge suppliers to reduce emissions
Tax on suppliers	Urges its emission reduction (number 12, 15) Do not urge its emission reduction (number 11, 14)

**Table 4:** The total negative impact of the supplier’s production on the environment.

Number	b	e_0_	Initial Situation	Downstream Enterprises Urged	Government Tax	Government Subsidy	Downstream Enterprise Urged + tax	Downstream Enterprise Urged + Subsidies
1	0.2	2200	2,371,224	2,344,441	\	2,227,238	\	2,227,238
2	0.2	2400	2,566,011	2,540,014	\	2,427,606	\	2,427,606
3	0.2	2600	2,756,788	2,731,567	\	2,623,853	\	2,623,853
4	0.2	2800	2,943,554	2,919,099	\	2,815,981	\	2,815,981
5	0.4	2200	2,118,330	2,080,552	\	2,010,333	\	2,010,333
6	0.4	2400	2,269,034	2,233,951	\	2,171,101	\	2,171,101
7	0.4	2600	2,411,656	2,379,190	\	2,323,301	\	2,323,301
8	0.4	2800	2,546,198	2,516,268	\	2,466,933	\	2,466,933
9	0.6	2200	1,877,711	1,840,939	\	1,804,367	\	1,804,367
10	0.6	2400	1,984,807	1,953,107	\	1,924,432	\	1,924,432
11	0.6	2600	2,079,628	2,052,729	2,019,675	2,030,997	\	2,030,997
12	0.6	2800	2,162,175	2,139,804	1,101,192	2,124,062	1,244,700	2,124,062
13	0.8	2200	1,645,981	1,618,653	\	1,604,348	\	1,604,348
14	0.8	2400	1,709,288	1,689,229	1,555,556	1,681,268	\	1,681,268
15	0.8	2600	1,755,935	1,742,497	305,556	1,739,079	509,447	1,739,079
16	0.8	2800	1,785,923	1,778,456	\	1,777,778	\	1,777,778

**Table 5:** Social welfare.

Number	b	e_0_	Initial Situation	Downstream Enterprises Urged	Government Tax	Government Subsidy	Downstream Enterprise Urged + Tax	Downstream Enterprise Urged + Subsidies
1	0.2	2200	1,794,563	1,822,846	\	1,877,932	\	1,877,932
2	0.2	2400	1,523,691	1,551,160	\	1,603,958	\	1,603,958
3	0.2	2600	1,257,530	1,284,192	\	1,334,754	\	1,334,754
4	0.2	2800	996,080	121,952	\	1,070,320	\	1,070,320
5	0.4	2200	1,267,237	1,309,995	\	1,340,647	\	1,340,647
6	0.4	2400	979,757	1,019,618	\	1,046,934	\	1,046,934
7	0.4	2600	703,178	740,226	\	764,938	\	764,938
8	0.4	2800	437,500	471,820	\	493,040	\	493,040
9	0.6	2200	814,646	860,636	\	875,201	\	875,201
10	0.6	2400	525,072	565,365	\	576,573	\	576,573
11	0.6	2600	254,175	289,064	254,410	2,973,355	\	2,973,355
12	0.6	2800	1954	31,735	63,602	37,547	72,865	37,547
13	0.8	2200	434,919	475,468	\	480,437	\	480,437
14	0.8	2400	158,334	190,260	160,000	192,787	\	192,787
15	0.8	2600	90,062	−66,040	10,000	−65,137	11,932	−65,137
16	0.8	2800	3,100,271	−293,430	\	−293,333	\	−293,333

**Table 6:** The government’s choice.

Combination Type	Government’s Choice
Under the combination of number 11, 14	Tax on suppliers (Focus on environmental pollution);
	Subsidies to suppliers (Focus on industrial development and consumer welfare)
Under the combination of number 12, 15	Tax on suppliers
Under the combination of other numbers	Subsidies to suppliers

## Data Availability

The data presented in this study are available on request from the corresponding author.
